# Clinical outcomes and early- prognostic biomarkers of primary biliary cholangitis with ductopenia

**DOI:** 10.3389/fimmu.2025.1680942

**Published:** 2025-12-02

**Authors:** Wenjuan Wang, Guo Zhang, Hui Liu, Lingna Lv, Jing Chang, Zefeng Liu, Huilan Ye, Huiguo Ding, Ying Han

**Affiliations:** 1 Department of Gastroenterology, Guangxi Hospital Division of The First Affiliated Hospital, Sun Yat-Sen University, Nanning, Guangxi, China; 2 Department of Gastroenterology and Hepatology, Laboratory for Clinical Medicine, Beijing You’an Hospital, Capital Medical University, Beijing, China; 3 Department of Pathology, Laboratory for Clinical Medicine, Beijing You`an Hospital, Capital Medical University, Beijing, China

**Keywords:** primary biliary cholangitis, ductopenia, clinical features, prognosis, biomarkers, alkaline phosphatase, antinuclear antibodies, gamma-glutamyl transferase

## Abstract

**Background:**

Ductopenia drives biochemical failure and histological progression in primary biliary cholangitis (PBC), influencing its course and prognosis, but its prevalence, features, and prognosis remain unclear. This study aimed to characterize ductopenia in PBC and identify early predictive biomarkers.

**Methods:**

From August 2013 to April 2025, 518 of the biopsy-proven PBC patients were enrolled, analyzed for demographics, pathology, and clinical features, and grouped by ductopenia presence. 201 patients were followed until June 15, 2025, with liver-related adverse events (including TIPS, splenectomy with portosystemic shunt or portoazygous devascularization, liver failure, death, or liver transplantation) as endpoints. Kaplan-Meier and Cox regression assessed prognosis.

**Results:**

The overall proportion of patients with PBC and ductopenia was 56.76% (294/518), Notably, ductopenia was present in 24.83% (74/298) of patients with early-stage disease. Compared with the group without ductopenia, the ductopenia group showed significantly higher levels of cholestasis indicators (such as TBIL, ALP, GGT, and TBA) and autoantibodies (ANA, AMA anti-gp210), but significantly lower levels of liver synthetic function indicators (such as ALB and cholinesterase) and blood components (RBC, PLT, and HGB) (all *P*<0.05). The median follow-up time was 7.60 years (interquartile range: 5.80–9.20 years). The prevalence of liver-related adverse events was significantly higher in PBC patients with ductopenia than in those without (*P*<0.05). Cox regression analysis confirmed that ductopenia (HR=8.868, 95% CI: 1.135–69.307, *P*=0.037) was an independent risk factor for the occurrence of liver-related adverse events in patients with PBC. Multivariable logistic regression analysis identified that serum ANA(≥1:1000) (OR= 2.180, 95% CI: 1.261–3.769), elevated GGT (OR = 1.002, 95% CI: 1.001–1.003, *P*= 0.001) and TBIL (OR= 1.020, 95% CI: 1.005–1.035), lowed ALB (OR= 0.943, 95% CI: 0.896–0.993) as biomarkers for ductopenia in patients with early-stage PBC.

**Conclusions:**

Ductopenia is relatively common in patients with PBC, and its prevalence significantly increases with disease progression. Ductopenia was an independent risk factor for the occurrence of liver-related adverse events in patients with PBC. ANA(≥1:1000), TBIL, GGT, and ALB are early predictive biomarkers for ductopenia in patients with PBC.

## Graphical Abstract

**Figure:**
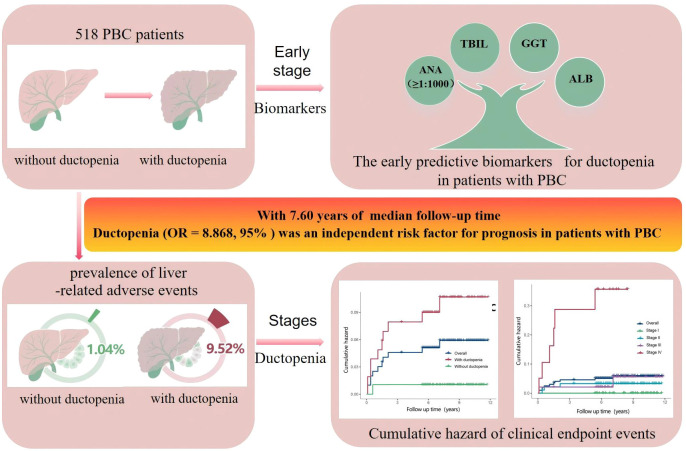
Receiver operating characteristic (ROC) curve for predicting early stage primary biliary cholangitis (PBC) with ductopenia. ALB, albumin; GGT, gamma-glutamyl transferase; ANA, antinuclear antibody.

## 1 Introduction

Primary biliary cholangitis (PBC) is a chronic autoimmune liver disease characterized by destructive granulomatous lymphocytic cholangitis, leading to progressive bile duct loss, cholestasis, fibrosis, and biliary cirrhosis. The global incidence and prevalence of PBC are on the rise (1–4), particularly in China (1, 5). The key mechanism underlying PBC involves the apoptosis of biliary epithelial cells that drive persistent damage to the bile ducts, which, in turn, accelerates liver fibrosis and ductopenia (6, 7). These concomitant pathological changes significantly complicate the diagnosis and treatment of PBC.

Patients with PBC primarily present with cholestasis, with the natural disease course showing individual variance (8) from asymptomatic slow progression to rapid deterioration (median duration: 10–15 years) (9). Bile duct reduction is common in PBC, and loss of bile ducts in >50% of portal tracts is defined as ductopenia (10). Ductopenia represents a significant bile duct lesion and cholestatic change in patients with PBC, as it can significantly increase the risk of cirrhosis and end-stage liver disease, ultimately leading to liver transplantation or death. It is one of the key features characterizing the poor biochemical response to ursodeoxycholic acid (UDCA) and progression of PBC (10–13). UDCA is the first-line treatment of PBC (10, 14, 15), but 30–40% of patients show poor response to the treatment (16–19) and experience a significantly worsened prognosis (20).

Early identification and intervention for ductopenia are crucial for blocking the progression of PBC to end-stage liver disease (21–23), and clarifying its early warning biomarkers is a key prerequisite to its diagnosis. Although liver histology serves as the gold standard for assessing ductopenia, its invasiveness limits its clinical application. Therefore, non-invasive biomarkers are urgently needed to achieve early prediction of ductopenia in patients with PBC.

However, current research on the clinical characteristics, prognostic impact, and early warning biomarkers of ductopenia in Chinese patients with PBC remains insufficient, and in-depth exploration is urgently needed. This study aimed to systematically reveal the clinical features of ductopenia in Chinese patients with PBC and its role in prognosis, explore biomarkers for early prediction of ductopenia, and provide evidence-based insights for improving patients’ survival.

## 2 Materials and methods

### 2.1 Study population

This retrospective study enrolled patients with biopsy-proven PBC, with no diagnosis of other types of hepatitis, from August 2013 to April 2025 at Beijing You`an Hospital, Capital Medical University. All patients received UDCA treatment at a dosage of 13–15 mg/kg. The studies involving human participants were reviewed and approved by the Ethics Committee of Beijing Youan Hospital, Capital Medical University(Jingyou Kelun Zi [2024] No. 020).Written informed consent to participate in this study was provided by the patient or patients’ legal guardian.

PBC diagnosis was based on meeting at least two of the following three criteria specified in the 2022 Clinical Practice Guidelines by the Asian Pacific Association for the Study of the Liver (10): (a) biochemical evidence of cholestasis indicated by elevated alkaline phosphatase (ALP) and gamma-glutamyl transpeptidase (GGT), with the exclusion of extrahepatic biliary obstruction by imaging studies; (b) presence of antimitochondrial antibody (AMA) or other PBC-specific antinuclear antibodies (ANA), including anti-sp100 antibody (anti-sp100) or anti-gp210 antibody (anti-gp210); and (c) histologic evidence of nonsuppurative destructive cholangitis mainly affecting the interlobular bile ducts.

The exclusion criteria were (1) PBC concurrent with other chronic liver diseases, such as viral hepatitis, autoimmune hepatitis, or alcoholic liver disease; (2) hepatocellular carcinoma and other malignant tumors; and (3) portal tract number of the liver tissue <11.

The core purposes of liver biopsy for PBC patients are to confirm the diagnosis and assess the condition, which include the followings: (1) Clarifying histological staging: Even in AMA-positive patients, some have atypical clinical manifestations (such as mild elevation of ALP/GGT, asymptomatic). Liver biopsy can accurately distinguish between Ludwig Stage I/II (early stage) and Stage III/IV (advanced stage); (2)Ruling out overlapping syndromes: For patients with concurrent strong positive ANA (≥1:1000) or significant elevation of ALT (>2×ULN), liver biopsy can rule out PBC-autoimmune hepatitis overlap; (3)Assessing liver damage before treatment: It provides pathological evidence for the evaluation of UDCA treatment response (e.g., the relationship between the degree of baseline bile duct loss and subsequent treatment response).

### 2.2 Data collection

Demographic, laboratory, histologic, and clinical data at baseline and at the time of liver biopsy (within 1 week of the liver biopsy) were collected. Laboratory data included biomarkers of hepatitis B and C; complete blood counts; liver biochemistry; coagulation function parameters; immunoglobulin levels; AMA, including AMA subtype M2 (AMA-M2); and ANA, including anti-gp210 and anti-sp100 antibody(anti-sp100). Histologic data included stages and degree of bile duct reduction. Clinical data included complications of portal hypertension (esophagogastric variceal bleeding, ascites, and hepatic encephalopathy) and edema. Follow-up data were mainly obtained by review of medical records and partly by telephone interview. The follow-up duration was defined as the interval between the date of diagnosis and the last visit or date of clinical outcome.

### 2.3 Histologic assessment

Percutaneous liver biopsy was performed under ultrasound guidance. The liver biopsy specimen measured ≥1.5 cm in length. The specimen was fixed in 10% formaldehyde solution, dehydrated with ethanol, embedded in paraffin, sectioned, and stained with hematoxylin-eosin, cytokeratin 7, and reticular fiber. The specimen was then observed under a light microscope. Two pathologists conducted a blinded review of the slides for histological diagnosis and staging. The two pathologists also evaluated the degree of bile duct loss. The presence of ductopenia was defined by the absence of bile ducts in more than 50% of portal tracts in a biopsy specimen containing more than 10 portal tracts.

Two extensive liver pathologists jointly reassessed all the liver specimens. In a double-blind situation, pathologists and hepatologists provided histological diagnosis and clinical information independently. In cases of disagreement between the pathologic diagnosis and clinical information, the pathologists and hepatologist reached a consensus through in-depth discussions.

### 2.4 Laboratory measurements

1. Liver biochemistry and coagulation function parameters: Serum liver biochemical parameters, including total bilirubin (TBIL, normal value: 5–21 μmol/L), direct bilirubin (normal value: <7 μmol/L), alanine transaminase (ALT, normal value: 9–50 U/L), aspartate transaminase (normal value: 15–40 U/L), gamma-glutamyl transpeptidase (GGT, normal value: 10–60 U/L), alkaline phosphatase (ALP, normal value: 50–135 U/L), albumin (ALB, normal value: 40–55 g/L), and total bile acid (normal value: <10 μmol/L), were measured using an automatic biochemical analyzer (ADVIA2400, Siemens). The coagulation function parameters included prothrombin time (normal value: 9.9–12.8 s) and prothrombin activity (normal value: 80–120%) and were measured by turbidimetry (ACL TOP, Werfen).

2. Immunoglobulins (Ig): These included IgG (normal value: 7.0–16.0 g/L), IgA (normal value: 0.7–4.0 g/L), and IgM (normal value: 0.4–2.3 g/L) were determined using immunoscatter turbidimetry.

3. Complete blood counts: White blood cells (normal value: 4–10×10^9^/L), absolute neutrophil count (normal value: 1.8–6.3×10^9^/L), hemoglobin (120–160 g/L for males, 115–150 g/L for females), and platelet count (normal value: 125–350×10^9^/L) were determined using a hematology analyzer (Sysmex XE-2100, Sysmex Corporation, Japan).

4. Autoantibodies: ANA (normal value:<1:100) and AMA (normal value:<1:100) were detected by indirect immunofluorescence. The AMA-M2 (normal value: <25RU/mL) was detected by enzyme linked immunosorbent assay (Ortho Clinical Diagnostics, Germany). Anti-sp100 and anti-gp210 were detected by immunoblotting(YHLO-Tenfly Phoenix-A, ShenZhen, China). Autoantibody detection reliability is ensured by standardized protocols: methods are conducted per kit instructions and laboratory SOPs, and results are double-blind interpreted by 2 experienced physicians.

### 2.5 Diagnostic criteria

Cirrhosis was defined according to the revised diagnostic criteria proposed by the Chinese Society of Hepatology (24). Decompensated cirrhosis was defined as the occurrence of ascites, esophageal variceal bleeding, or hepatic encephalopathy. In addition, the diagnosis and staging of PBC was based on the Ludwig criteria (25). Early-stage (Stage I, Stage II) PBC was indicated by portal non-suppurative destructive cholangitis, whereas advanced-stage (Stage III, Stage IV) PBC was indicated by fibrosis or cirrhosis.

### 2.6 Definition of clinical endpoint

The clinical endpoint was the prevalence of liver-related adverse events—esophagogastric variceal bleeding, refractory ascites, transjugular intrahepatic portosystemic shunt, splenectomy combined with portosystemic shunt or devascularization, liver failure, clinical death related to liver disease, or liver transplantation—as of June 15, 2025.

The duration of follow-up was defined as the interval between the date of diagnostic liver biopsy and the last visit or the date of clinical outcome. The starting date was defined as the date of histological diagnosis of the liver specimen in patients with PBC. Loss to follow-up was defined as the failure to follow the patients for 6 consecutive months or loss of contact.

### 2.7 Statistical analysis

All data were statistically analyzed using IBM SPSS 27.0. Continuous variables are presented as mean ± standard deviation or median (interquartile range), as appropriate. Categorical variables are shown as counts and percentages. Chi-square test or Fisher exact test was used to compare categorical variables, whereas the Mann–Whitney U-test or t-test was used to compare the continuous variables. Logistic regression analysis was used to identify the risk factors for ductopenia in patients with PBC. Kaplan–Meier curves and Cox proportional hazards model were used to evaluate the predictive value of baseline clinical characteristics for the long-term prognosis of patients with PBC and ductopenia. Receiver operating characteristic curves were plotted to evaluate the diagnostic value of various parameters in identifying ductopenia in patients with early-stage PBC. *P* value <0.05 was considered statistically significant.

## 3 Results

### 3.1 General characteristics of patients with PBC

**Figure 1:**
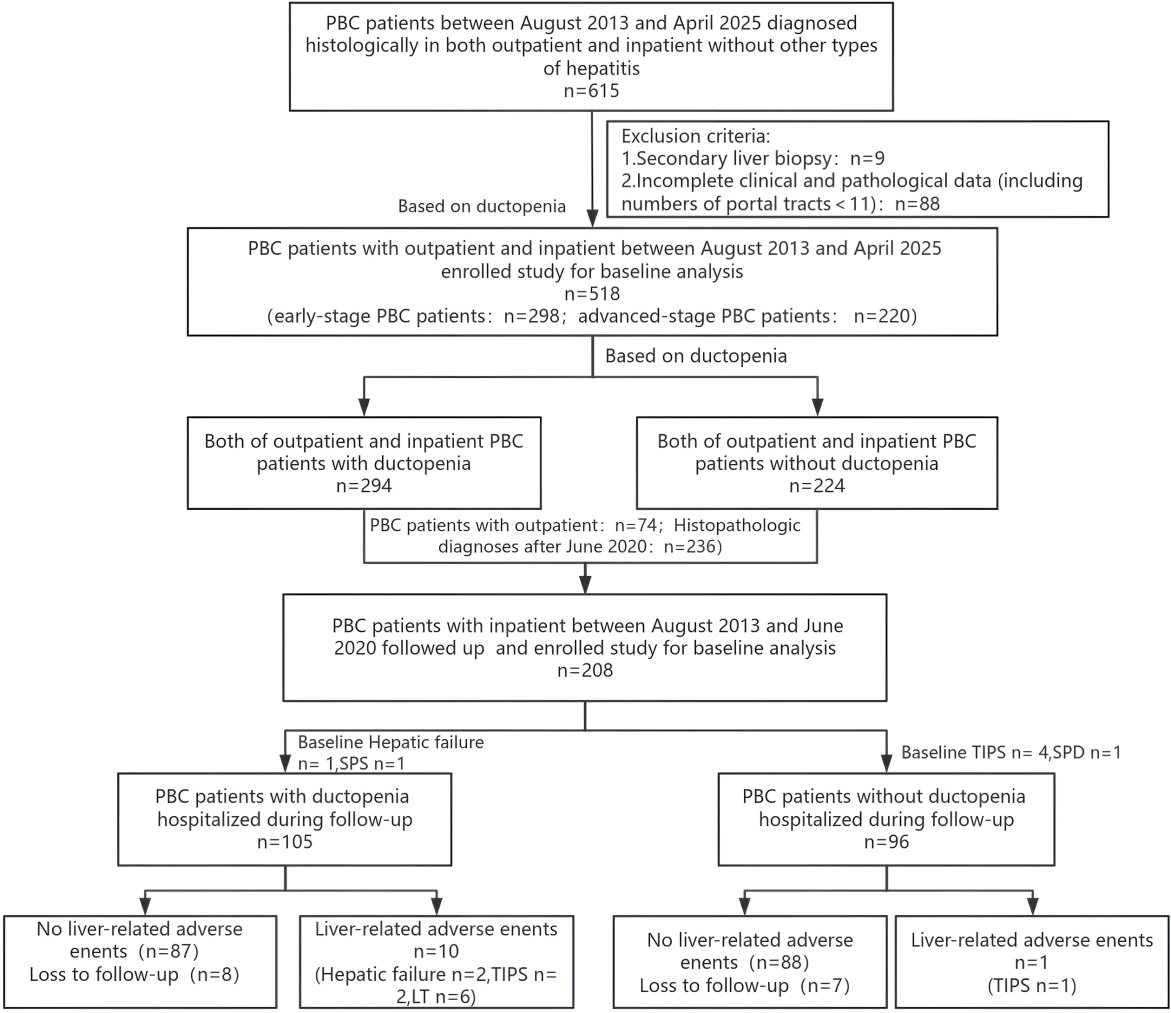
Flow-chart of study enrolment of patients with primary biliary cholangitis (PBC).

A total of 518 patients with biopsy-proven PBC were enrolled. Among PBC patients, 107 cases (20.66%) were serum AMA-negative, of which 25 were positive for M2 (23.36%, 25/107); 57 cases (11.00%) were serum ANA-negative, with 3 positive for gp210 and 4 positive for SP100; only 9 patients (1.74%) were negative for both AMA and ANA. Based on the Ludwig criteria, 298 patients had early-stage PBC (with ductopenia vs. without ductopenia: 74 vs. 220) and 220 patients had advanced-stage PBC. Patients with PBC were categorized into those with ductopenia (n = 294) and without ductopenia (n = 220). Furthermore, 208 hospitalized patients with biopsy-proven PBC between August 2013 and June 2020 were followed-up. As of June 15, 2025, seven patients with endpoint events at baseline were excluded, and finally 201 patients with PBC (including 15 lost to follow-up) were included in the follow-up analysis (**Figure 1**).

### 3.2 Baseline characteristics of patients with ductopenia

The baseline demographic and laboratory data are shown in **Table 1**. The median age was 50.81 years, and 453 (87.45%) were female. According to the Ludwig staging system, 97 patients (18.73%) were in stage I, 201 (38.80%) in stage II, 159 (30.69%) in stage III, and 61 (11.78%) in stage IV.

**Table 1:** Baseline characteristics of patients with PBC.

Characteristics	Overall PBC (n=518)	PBC with ductopenia (n = 294)	PBC without ductopenia (n =224)	T/ χ^2^/Z-value	*P*-value
Female	453 (87.45%)	253 (86.05%)	200 (89.28%)	1.210	0.271
Age (years, `X ± s)	50.81 ± 10.39	51.28 ± 10.55	50.18 ± 10.16	-1.190	0.234
IgA (g/L)	2.93 (2.29, 3.89)	3.39 (2.38,4.15)	2.84 (2.21,3.52)	-3.979	<0.001
IgG (g/L)	16.49 ± 5.44	16.82 ± 6.25	16.08 ± 4.11	-1.539	0.124
IgM (g/L)	3.00 (1.81, 4.09)	3.52 (2.04,4.36)	2.65(1.53,3.70)	-4.053	<0.001
PT (s)	16.39 ± 2.60	16.68 ± 2.80	16.02 ± 2.28	-2.877	0.004
PTA (%)	99.70 ± 19.93	96.90 ± 22.87	103.40 ± 14.50	3.981	<0.001
TBIL (μmol/L)	19.00 (13.08-32.73)	26.75 (16.40,52.00)	14.60 (11.10,10.40)	-10.049	<0.001
DBIL (μmol/L)	7.65 (4.30,19.80)	10.90 (5.60,30.70)	5.30 (3.50,10.50)	-7.689	<0.001
CHE (U/L)	6155.00 ± 2120.00	4537.00 ± 1994.00	6835.00 ± 2093.00	6.628	<0.001
GGT (U/L)	157.00 (65.00,291.00)	209.00 (84.00,381.00)	107.50 (51.00,207.50)	-5.623	<0.001
ALT (U/L)	46.00 (25.00,77.00)	49.00 (26.00,85.00)	43.00 (22.00,63.00)	-2.753	<0.001
AST (U/L)	50.00 (32.00,90.25)	64.00 (38.00,100.00)	39.00 (27.00,58.00)	-6.607	<0.001
TBA (U/L)	20.00 (6.70,53.70)	33.75 (12.60,77.70)	9.70 (4.40,22.20)	-8.928	<0.001
ALB (g/L)	40.25 ± 6.61	38.24 ± 6.24	42.90 ± 6.14	8.507	<0.001
ALP (U/L)	173.00 (107.00,280.0)	216.50 (136.50,343.50)	132.50 (91.00,189.50)	-7.365	<0.001
NEUT (×10^9^/L)	2.93 (2.21,3.86)	2.88 (2.12,4.01)	3.09 (2.42,3.81)	-1.127	0.260
WBC (×10^9^/L)	5.80 ± 2.55	5.66 ± 2.90	5.99 ± 1.99	1.553	0.121
RBC (×10^12^/L)	3.99 ± 0.73	3.75 ± 0.76	4.31 ± 0.55	9.619	<0.001
PLT (×10^9^/L)	198.12 ± 85.88	176.00 ± 86.00	227.00 ± 76.00	7.114	<0.001
HGB (g/L)	119.55 ± 21.88	112.96 ± 22.33	`128.20 ± 17.95	8.609	<0.001
ANA antibody				22.514	<0.001
Negative(<1:100)	57(11.00%)	33(11.22%)	24(10.72%)		
Non-Strong Positive (1:100 ≥ antibody titer < 1:1000)	230(44.40%)	105 (35.71%)	125 (55.80%)		
Strong Positive (≥ 1:1000)	231 (44.59%)	156 (53.06%)	75 (33.48%)		
AMA antibody				7.578	0.023
Negative(< 1:100)	107(20.66%)	59(20.07%)	48(21.43%)		
Non-Strong Positive (1:100 ≥ antibody titer < 1:1000)	116(22.39%)	54 (18.37%)	62 (27.68%)		
Strong Positive (≥ 1:1000)	295 (56.95%)	181 (61.56%)	114 (50.89%)		
Anti-gp210 antibody				28.884	<0.001
Non-Strong Positive (-/+/++/+++)	454 (87.64%)	247 (84.01%)	207 (92.41%)		
Strong Positive (++++)	64 (12.36%)	47 (15.99%)	17 (7.59%)		
AMA-M2 antibody				2.327	0.127
Non-Strong Positive (<800 RU/mL)	385 (74.32%)	211 (71.77%)	174 (77.67%)		
Strong Positive (≥800 RU/mL)	133 (25.68%)	83 (28.23%)	50 (22.32%)		

Continuous variables are presented as median (interquartile range) or mean (standard deviation). Categorical variables are shown as counts and percentages.

PBC, primary biliary cholangitis; IgA, immunoglobulin A; IgG, immunoglobulin G; IgM, immunoglobulin M; PT, prothrombin time; PTA%, prothrombin time activity; TBIL, total bilirubin; DBIL, direct bilirubin; CHE, cholinesterase; GGT, gamma-glutamyl transferase; ALT, alanine aminotransferase; AST, aspartate aminotransferase; TBA, total bile acid; ALB, albumin; ALP, alkaline phosphatase; WBC, white blood cell; RBC, red blood cell, NEUT, neutrophil count; PLT, platelet count; HGB, hemoglobin; ANA, antinuclear antibody; AMA, antimitochondrial antibody.

Compared with those without ductopenia, patients with PBC and ductopenia had significantly higher levels of alanine transaminase (median: 49.00 vs 43.00U/L), aspartate transaminase (median: 64.00 vs 39.00 U/L), TBIL (median: 26.75 vs 14.60 μmol/L), direct bilirubin (median: 10.90 vs 5.30 μmol/L), total bile acid (median: 33.75 vs 9.70 μmol/L), ALP (median: 216.50 vs 132.50 U/L), GGT (median: 209.00 vs 107.50 U/L), IgM (median: 3.52 vs 2.65 g/L), ANA, AMA, and Anti-gp210, as well as longer prothrombin time (mean: 16.68 vs 16.02 s) (all *P* values < 0.05). Conversely, patients with PBC and ductopenia had significantly lower levels of ALB (mean: 38.24 vs 42.90 g/L), cholinesterase (median: 4537.00 vs 6835.00 U/L), prothrombin activity (mean: 96.90 vs 103.40%), RBC (mean:3.75 vs 4.31×10^12^/L), PLT (mean:176.00 vs 227.00 ×10^9^/L), and hemoglobin (mean: 112.96.00 vs 128.20×10^9^g/L) (all *P* values < 0.05).

### 3.3 Baseline factors associated with outcomes

The baseline characteristics of patients with PBC included in the follow-up analysis (n = 201) are shown in **Supplementary Table 1**. The maximum follow-up duration was 11.80 years, with a median follow-up duration of 7.60 years (interquartile range: 5.80–9.20). Among the 201 patients followed up in this study, 11 cases (5.47%) experienced liver-related adverse events, including 2 cases of liver failure,3 cases treated with transjugular intrahepatic portosystemic shunt, and 6 cases of liver transplantation(LT). In the PBC subgroup with ductopenia, 10 liver-related adverse events were recorded, including 2 cases of liver failure, 2 cases of TIPS, and 6 cases of liver transplantation. In the PBC subgroup without ductopenia, the liver-related adverse event occurred, which was a case of TIPS.

Univariate Cox regression analysis showed that the following factors were significantly associated with prognosis: anti-gp210 antibody (hazard ratio [HR] = 1.409, 95% confidence interval [CI]: 1.017–1.957, *P* = 0.039), ductopenia (HR = 9.603, 95% CI: 1.229–75.003, *P* = 0.031), alanine transaminase (HR = 1.004, 95% CI: 1.001–1.007, *P* = 0.005), Ludwig stages(III/IV vs I/II) (HR = 5.520, 95% CI:1.464-20.815, *P* = 0.012)and cholinesterase (HR = 1.000, 95% CI: 0.999–1.000, *P* = 0.020). On multivariate Cox regression analysis, ductopenia (HR = 8.868, 95% CI: 1.135–69.307, *P* = 0.037) was the only independent risk factor for liver-related adverse events (**Table 2**).

**Table 2:** Univariate and multivariate logistic regression analysis to identify baseline factors associated with PBC.

Characteristics	Univariate analysis		Multivariate analysis	
	Hazard ratio(95% CI)	*P*-value	Hazard ratio(95% CI)	*P*-value
Anti-gp210	1.409 (1.017–1.957)	0.039		
CHE(U/L)	1.000 (0.999–1.000)	0.020		
ALT(U/L)	1.004 (1.001–1.007)	0.005		
Ludwig stages (III/IV vs I/II)	5.520(1.464-20.815)	0.012		
Ductopenia	9.603 (1.229–75.003)	0.031	8.868 (1.135–69.307)	0.037

PBC, primary biliary cholangitis; CI, confidence interval; HR, hazard ratio; CHE, cholinesterase; ALT, alanine aminotransferase

**Figure 2:**
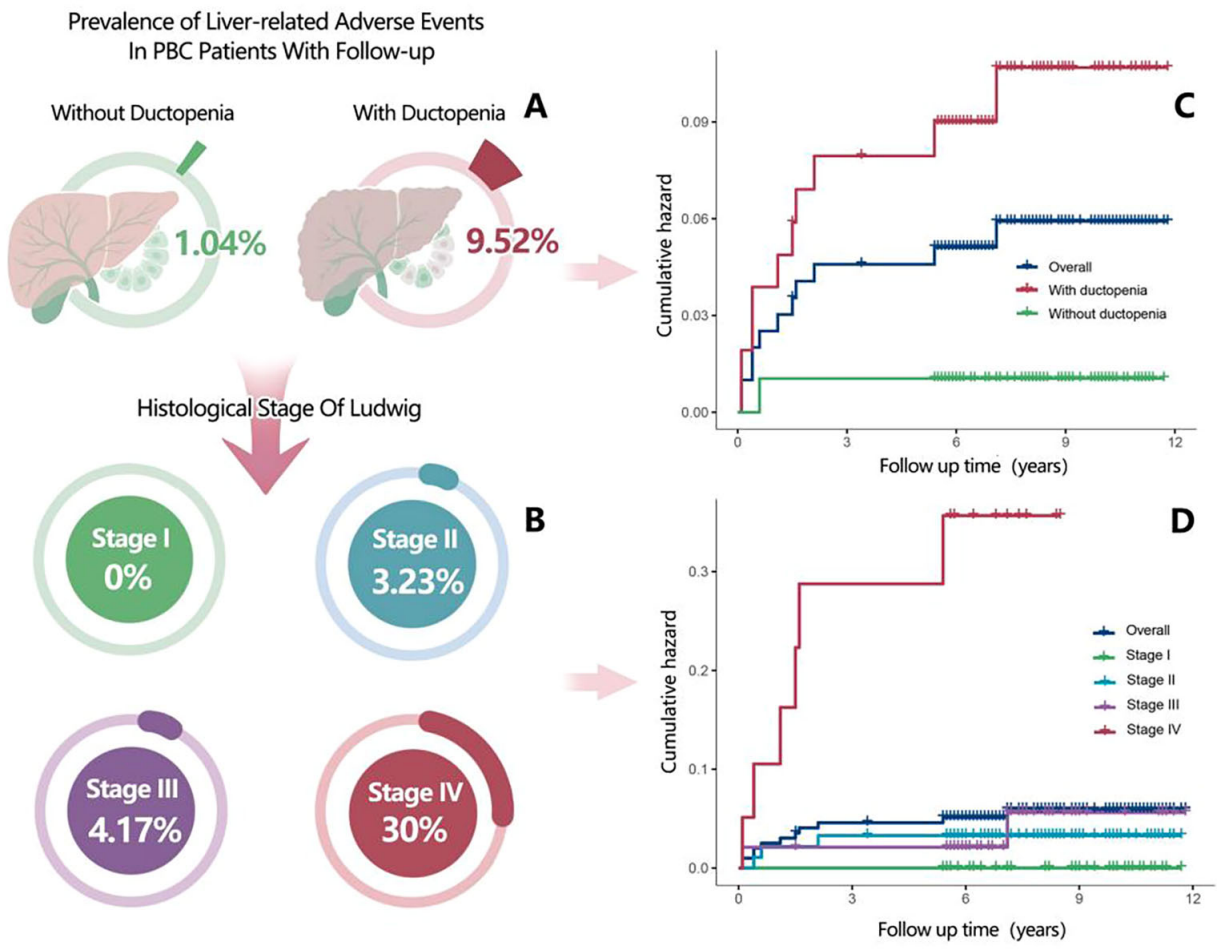
**(A)** Prevalence of liver-related adverse events in patients with primary biliary cholangitis (PBC) (with ductopenia vs without ductopenia) who were included in the follow-up analysis. **(B)** Prevalence of liver-related adverse events in patients with PBC according to Ludwig staging. **(C)** Kaplan–Meier plots for cumulative incidence of liver-related adverse events in patients with PBC (with ductopenia vs without ductopenia). **(D)** Kaplan–Meier plots for cumulative incidence of liver-related adverse events in patients with PBC according to histologic stages during follow-up.

When grouped by ductopenia status, the incidence of liver-related adverse events was significantly higher in the ductopenia group than in the non-ductopenia group (9.52% [10/105] vs. 1.04% [1/96]) (**Supplementary Table 2**; **Figures 2A, C**). When grouped by Ludwig stage, the prognosis of patients with PBC worsened with the progression of Ludwig stages. No liver-related adverse events were observed in patients with Stage I PBC. The prevalence of liver-related adverse events was 3.23% (3/93) in patients with Stage II PBC, 4.17% (2/48) in those with Stage III PBC, and 30.00% (6/20) in those with Stage IV PBC (**Supplementary Table 3**; **Figures 2B, D**).

Stratified by ductopenia and Ludwig stage revealed that among patients with ductopenia, theincidence of liver-related adverse events was 5.4% (2/37) in stage II, 4.2% (2/48) in stage III, and 30% (6/20) in stage IV (**Supplementary Figure 4A**). Similarly, when stratifying by ductopenia and cirrhosis status, the incidence was 4.7%(4/85) in non-cirrhotic patients with ductopenia, contrasting with 30% (6/20) in cirrhotic patients with ductopenia (**Supplementary Figure 4B**).

Further subgroup analysis showed: the incidence of liver-related adverse events was 1.04% (1/96) in the “early-stage PBC without ductopenia” subgroup, and 5.41% (2/37) in the “early-stage PBC with ductopenia” subgroup (slightly higher than the ductopenia-free early-stage subgroup). Notably, there were no patients in the “advanced-stage PBC without ductopenia” subgroup—consistent with PBC’s pathological progression, as long-term bile duct damage in advanced-stage PBC (Stage III/IV) causes high ductopenia rates, and all advanced-stage PBC patients in our cohort had ductopenia on histology. In contrast, the incidence of liver-related adverse events was 11.76% (8/68)in the “advanced-stage PBC with ductopenia” subgroup, significantly higher than that in the two early-stage subgroups (**Supplementary Figure 5**).

### 3.4 Biochemical indicators associated with ductopenia in early-stage PBC

Baseline characteristics of patients with early-stage PBC are shown in **Supplementary Table 4**. Univariate logistic analysis showed significant differences in IgM, ANA (≥1:1000), ALP, GGT, TBIL, TBA, and ALB levels between early-stage PBC patients with and without ductopenia(*P* < 0.05, **Supplementary Table 5**). After adjusting for potential confounders, multivariate analysis confirmed that ANA ≥1:1000 (odds ratio [OR] = 2.180, 95% CI: 1.261–3.769), elevated GGT (OR = 1.002, 95% CI: 1.001–1.003, *P* = 0.001), elevated TBIL (OR = 1.020, 95% CI: 1.005–1.035), and lower ALB (OR = 0.943, 95% CI: 0.896–0.993) were independent associated with ductopenia (*P* < 0.05, **Supplementary Table 5**). Based on these four variables, we developed a novel risk score model for ductopenia in early-stage PBC as follows: Risk Score = 0.195 + 0.774× (1 if ANA≥1:1000,else 0)+0.020×TBIL(μmol/L)+0.002×GGT(U/L)-0.059×ALB(g/L).

## 4 Discussion

The present study systematically investigated the clinical characteristics, prognostic impact, and early warning biomarkers of ductopenia in patients with PBC. Our findings showed that ductopenia was relatively common in patients with PBC, and its prevalence increased significantly with disease progression. Ductopenia was not only a key histological feature of PBC progression but also an independent risk factor for long-term prognosis, including liver-related adverse events. The study identified ANA (≥1:1000), TBIL, GGT, and ALB as biomarkers of ductopenia in early-stage PBC.

In our study, the proportion of ductopenia in patients with PBC was 56.76%, of whom 24.83% had early-stage PBC (Stages I and II), comparable to the 25.53% reported by Yu et al. (26). Furthermore, ductopenia was more prevalent in patients with advanced-stage PBC (Stages III and IV), consistent with the cumulative effect of bile duct injury (26, 27). The high rate of ductopenia may be due to selection bias, as liver biopsies are recommended mainly for AMA-negative PBC or PBC with poor response to UDCA. These findings suggest that in addition to the known immune disorder factors, ductopenia may be an independent risk for inadequate response to UDCA.

The multivariate logistic analysis identified ANA (≥1:1000), GGT, TBIL, and ALB as potential biomarkers for ductopenia in patients with early-stage PBC. The four potential biomarkers for ductopenia play different roles in its pathogenesis.

The elevation of GGT reflects damage and compensatory hyperplasia of bile duct epithelial cells, while the elevation of TBIL directly indicates bile excretion disorders. Specifically, immune attack, CD4^+^T cell-mediated inflammation, abnormal autophagy, and imbalance of miR-506 trigger the apoptosis of bile duct epithelial cells, resulting in reduced bile ducts and cholestasis (28–30). Chronic inflammation inhibits the FXR pathway, leading to the accumulation of hydrophobic bile acids (31), and mediates cell death and fibrosis, creating a vicious cycle of cholestasis (29, 32). In addition, impaired biliary bicarbonate secretion enhances the toxic effects of bile acids on the bile ducts and promotes the release of GGT (21). Further, activation of hepatic progenitor cells and the Notch pathway leads to overexpression of GGT in newly formed bile duct cells (33), and intestinal flora imbalance accelerates disease progression (34– 35). Together, GGT and TBIL serve as serological markers of ductopenia, indicating the rationality of their use as early markers.

In addition, ANA (≥1:1000) and ALB had significant value as biomarkers of ductopenia in early-stage PBC. As a characteristic autoantibody of PBC, high-titer ANA is closely associated with bile duct damage caused by immune disorders (10). In our study, the positive rate of ANA (≥1:1000) was significantly increased in the ductopenia group than in the non-ductopenia group, suggesting that strong autoimmune activation may be a key driver of ductopenia. The immune activation may directly attack bile duct epithelial cells, leading to bile duct destruction and loss. High-titer ANA may reflect a higher disease activity, particularly a higher risk of bile duct damage. Consequently, chronic liver injury caused by bile duct loss gradually affects hepatocyte function, resulting in reduced ALB synthesis. ALB reflects a decline in liver synthetic function.

The combination of these four indicators could provide an early warning for ductopenia from multiple dimensions, such as immune activation, bile excretion, and liver synthetic function. In clinical practice, detection of abnormalities in the above biomarkers suggest a high possibility of ductopenia. In such cases, liver histological examination should be performed to clarify the degree of bile duct loss and guide the formulation of individualized treatment plans. For patients who are not eligible for or temporarily refuse liver biopsy, dynamic monitoring of these indicators can be performed. For example, a progressive increase in ANA, GGT, and TBIL, along with a continuous decrease in ALB, suggests an increased risk of bile duct loss progression. In such cases, the patient’s response to UDCA should be evaluated in a timely manner, and combination therapy with second-line drugs, such as obeticholic acid (10), or exploring innovative treatment regimens should be considered to delay disease deterioration.

In the prognostic analysis of a subgroup of 201 patients with PBC, multivariate Cox regression analysis showed that after adjusting for potential confounders, such as age and sex, ductopenia remained an independent predictor of poor prognosis in patients with PBC. The negative impact of ductopenia on prognosis was reflected in several aspects. First, the histological stage of the ductopenia group was significantly later, suggesting a more severe condition and higher risk of end-stage events. This study showed that among early-stage PBC patients, the incidence of liver-related adverse events was 5.41% (2/37) for those with ductopenia and 1.04% (1/96) for those without ductopenia. These patients of early onset ductopenia showed a trend toward worse outcomes, though the number of events was limited. Early identification for ductopenia are crucial for blocking the progression of PBC to end-stage liver disease (21–23). Second, during follow-up, the prevalence of adverse liver events in patients with ductopenia was significantly increased and was positively correlated with the progression of histological stage. This finding is consistent with the result of a previous longitudinal study (36) in which 83% (25/30) of patients with poor response to UDCA therapy (Toronto criteria) showed progressive fibrosis accompanied by ductopenia in the second liver biopsy after 10 years, and the prognosis of such patients was significantly deteriorated (HR = 4.2, 95%CI 2.1–8.3). Further, in the present study, the ductopenia group exhibited more severe cholestasis (elevated TBIL, ALP, GGT, and total bile acid) and poorer liver synthetic and reserve functions (decreased ALB, cholinesterase, and prothrombin activity), consistent with the results of a 30-year follow-up study in the United States (37).

As an independent prognostic factor, the core mechanism of ductopenia in PBC lies in driving biochemical response failure and histological progression (including fibrosis) (14). Clinically, despite regular UDCA administration, bile duct damage in patients with ductopenia is difficult to improve (38). Histological analyses often show that ductopenia and fibrosis coexist in patients with PBC, and the two form a mutually reinforcing vicious cycle—ductopenia initiates hepatic fibrosis, while the aggravation of fibrosis exacerbates ductopenia (26). Together, they promote disease progression and constitute the core pathological factors determining the prognosis of PBC (39, 40). This mechanism is supported by the incidence data of liver-related adverse events across subgroups: the incidence was 11.76% (8/68) in the subgroup of advanced-stage PBC with ductopenia, whereas it was only 1.04% (1/96) in the “early-stage PBC without ductopenia” subgroup and 5.41% (2/37) in the “early-stage PBC with ductopenia” subgroup. Such a notable difference in event incidence further confirms that the coexistence of advanced fibrosis and ductopenia exacerbates the risk of adverse outcomes in PBC patients, highlighting the critical prognostic role of this combined pathological feature.

This vicious cycle directly leads to the disease chain of “bile duct absence → fibrosis → portal hypertension → end-stage events.” In our study, the prevalence of portal hypertension complications (esophagogastric variceal bleeding/ascites) in the ductopenia group was significantly higher, which may explain why 34% of patients with non-hepatitis cirrhosis in a previous study developed portal hypertension early (hepatic venous pressure gradient, > 12 mmHg (39–41). Moreover, multivariate analysis confirmed that ductopenia independently predicted poor prognosis. As an “initiator” of fibrosis progression, ductopenia is not only associated with poor response to UDCA and low transplant-free survival rate, but is a core indicator of disease progression and intervention timing. Advanced fibrosis (stages 3–4) and poor response to UDCA are the best indicators of transplant-free survival rate and whether ALP and TBIL can return to normal after treatment (41, 42).

Immune-mediated chronic destructive cholangitis is one of the core mechanisms underlying ductopenia in patients with PBC (10, 14, 15). The present study showed that ductopenia was associated with unique immune disorders, characterized by elevated IgM (indicating disease progression and poor prognosis) (43) and decreased IgG, consistent with previous reports (26, 44). Immune markers, such as AMA, ANA, and anti-gp210, showed a high positive rate in the ductopenia group, with the AMA negative rate reaching 20.66%, which is significantly higher than the 5–10% positive rate reported in the literature (10, 31). This discrepancy may be related to a selection bias arising from inclusion of AMA-negative/UDCA non-responders in liver histological examinations. Notably, AMA-negative patients suffer from more severe bile duct damage and have a poorer prognosis (45). Follow-up data indicated that positive anti-gp210 could significantly predict adverse liver events. In addition, known risk factors such as female sex and advanced age (10, 14, 15) showed no difference between the ductopenia and non-ductopenia groups, suggesting that ductopenia, as an independent prognostic factor, can be a reliable marker of poor prognosis when traditional serum markers are negative.

This study has several limitations. First, as a single-center retrospective study, we could not accurately collect data on the patients’ compliance with UDCA treatment. Second, the cut-off values of the four markers may be affected by the characteristics of the single-center population. Third, despite being China’s largest PBC-ductopenia cohort, its retrospective design is limited; more critically, only 11 (5.47%) of 201 followed patients had liver-related adverse events, restricting multivariate Cox regression power and raising overfitting risk. Future research should focus on the following aspects: (1) conduct multi-center prospective studies to verify biomarkers and reduce overfitting, while increasing liver elastography indicators; (2) intensively study the specific molecular mechanisms underlying the effect of bile duct absence on the prognosis of PBC, providing a theoretical basis for the development of new therapeutic targets; and (3) explore a clinical decision support system using the present study results to achieve individualized risk assessment and precise treatment for patients with PBC.

In conclusion, our study emphasized the important role of ductopenia in the prognosis of PBC, suggesting that doctors should incorporate ductopenia into routine assessments, dynamically integrate histological information (stage/degree of ductopenia) with serum biological markers (GGT, TBIL, ALB, and ANA), comprehensively evaluate the patients’ disease status and progression risk, and optimize the management of patients with PBC. Closer monitoring for ductopenia should be paid to patients with PBC with a high-titer ANA, elevated GGT and TBIL, or decreased ALB. When necessary, active liver biopsy should be performed to timely initiate or adjust treatment strategies, delay the progression of liver fibrosis, prevent complications of portal hypertension, reduce the need for liver transplantation, and ultimately improve the quality of life and long-term prognosis of patients with PBC.

## Data Availability

The raw data supporting the conclusions of this article will be made available by the authors, without undue reservation.

## Supplementary material

Supplementary Figure 4 Cumulative Hazard of Liver-Related Adverse Events by in Primary Biliary Cholangitis with Ductopenia. Stratification by (A) Stages, (B) with cirrhosis.
