# Correction: Clinical outcomes and early- prognostic biomarkers of primary biliary cholangitis with ductopenia

**DOI:** 10.3389/fimmu.2026.1828569

**Published:** 2026-03-24

**Authors:** Wenjuan Wang, Guo Zhang, Hui Liu, Lingna Lv, Jing Chang, Zefeng Liu, Huilan Ye, Huiguo Ding, Ying Han

**Affiliations:** 1 Department of Gastroenterology, Guangxi Hospital Division of The First Affiliated Hospital, Sun Yat-Sen University, Nanning, Guangxi, China; 2 Department of Gastroenterology and Hepatology, Laboratory for Clinical Medicine, Beijing You’an Hospital, Capital Medical University, Beijing, China; 3 Department of Pathology, Laboratory for Clinical Medicine, Beijing You`an Hospital, Capital Medical University, Beijing, China

**Keywords:** primary biliary cholangitis, ductopenia, clinical features, prognosis, biomarkers, alkaline phosphatase, antinuclear antibodies, gamma-glutamyl transferase

An incorrect number was provided for the “Natural Science Foundation Project of Guangxi”. The correct number is “[2024GXNSFAA010020]”.

The original version of this article has been updated.

