# Supplementary material for: Clinical outcomes and early- prognostic biomarkers of primary biliary cholangitis with ductopenia
Source: Front Immunol. 2025 Dec 2;16:1680942. doi: 10.3389/fimmu.2025.1680942 (PMC12705372; doi:10.3389/fimmu.2025.1680942)
Supplement: Supplementary Figure 4 — Cumulative Hazard of Liver-Related Adverse Events by in Primary Biliary Cholangitis with Ductopenia. Stratification by (A) Stages, (B) with cirrhosis. [file Table1.docx]

Supplementary Material

# Supplementary Tables

**Supplementary Table 1**. Baseline characteristics of hospitalized patients with PBC included the follow-up analysis

| **Characteristics** | **Overall PBC cases**  **(n = 201)** | **PBC with ductopenia**  **(n = 105)** | **PBC without ductopenia**  **(n = 96)** | **T/**  **χ^2^/Z -value** | ***P -*value** |
| --- | --- | --- | --- | --- | --- |
| Female | 179 (89.05%) | 93 (88.57%) | 86 (89.58%) | 0.053 | 0.818 |
| Age (years, ‾X±s) | 49.59±9.34 | 49.71±9.29 | 49.45±9.38 | -0.202 | 0.840 |
| IgA (g/L) | 3.02(2.29,4.05) | 3.39(2.38,4.17) | 2.87(2.22,3.72) | -1.970 | 0.049 |
| IgG (g/L) | 17.23±4.96 | 17.96±5.35 | 16.44±4.39 | -2.193 | 0.029 |
| IgM (g/L) | 3.58 (2.11,5.21) | 3.76 (2.19,5.33) | 3.15 (2.00,4.99) | -1.605 | 0.109 |
| CHE (U/L) | 6227.50±2299.90 | 5962.00±2148.00 | 6518.00±2433.50 | 1.720 | 0.087 |
| GGT (U/L) | 194.00 (81.00,352.50) | 235.00 (89.50,454.00) | 167.00 (73.50,288.00) | -2.378 | 0.017 |
| ALT (U/L) | 48.00 (28.00,71.50) | 49.00 (27.00,76.00) | 46.00 (28.50,65.50) | -0.653 | 0.514 |
| ALP (U/L) | 191.00 (118.00,282.00) | 228.00 (150.00,334.00) | 147.50 (97.50,221.00) | -3.7359 | <0.001 |
| ANA antibody |  |  |  | 6.123 | 0.013 |
| Non-Strong Positive  (<1:1000) | 101 (50.25%) | 44 (41.90%) | 57 (59.38%) | 19.725 | <0.001 |
| Strong Positive  (≥1:1000) | 100 (49.75%) | 61 (58.09%) | 39 (40.62%) |  |  |
| AMA antibody |  |  |  | 0.592 | 0.442 |
| Non-Strong Positive  (<1:1000) | 72 (35.82%) | 35 (33.33%) | 37 (38.54%) |  |  |
| Strong Positive (≥1:1000) | 129 (64.18%) | 70 (66.67%) | 59 (61.46%) |  |  |
| Anti-gp210 antibody |  |  |  | 1.803 | 0.179 |
| Non-Strong Positive  (-/+/++/+++) | 149 (74.13%) | 82 (78.10%) | 67 (69.79%) |  |  |
| Strong Positive  (++++) | 52 (25.87%) | 23 (21.90%) | 29 (30.21%) |  |  |
| AMA-M2 antibody |  |  |  | 2.889 | 0.089 |
| Non-Strong Positive  (<800 RU/mL) | 90 (44.78%) | 53 (50.48%) | 37 (38.54%) |  |  |
| Strong Positive  (≥800 RU/mL) | 111 (55.22%) | 52 (49.52%) | 59 (61.46%) |  |  |
| Anti-sp100 antibody |  |  |  | 3.181 | 0.075 |
| Negative (-) | 173(86.07%) | 86(81.90%) | 87(90.62%) |  |  |
| Positive(+) | 28(13.93%) | 19(18.09%) | 9(9.38%) |  |  |
| Decompensated period | 30 (14.93%) | 25 (23.81%) | 5 (5.21%) | 13.665 | <0.001 |
| Ascites | 22 (10.95%) | 18 (17.14%) | 4 (4.17%) | 7.383 | 0.007 |
| esophagogastric variceal or bleeding | 33 (16.42%) | 30 (28.57%) | 3 (3.13%) | 21.846 | <0.001 |
| Follow-up duration (years) | 7.60 (5.80,9.20) | 7.10 (5.70,8.85) | 8.15 (6.10,9.90) | -2.234 | 0.025 |
| Liver-related adverse events | 11 (5.47%) | 10 (9.52%) | 1 (1.04%) | 5.431 | 0.020 |

PBC, primary biliary cholangitis; IgA, immunoglobulin A; IgG, immunoglobulin G; IgM, immunoglobulin M; CHE, cholinesterase; GGT, gamma-glutamyl transferase; ALT, alanine aminotransferase; ALP, alkaline phosphatase; ANA, antinuclear antibody; AMA, antimitochondrial antibody.

**Supplementary Table 2**. Liver-related adverse events of hospitalized patients with PBC with ductopenia at follow-up

| **Group** | **Number /Total number of events** | **Prevalence** | **Composition of Liver-Related Adverse Events** |
| --- | --- | --- | --- |
| With ductopenia | 10/105 | 9.52% | Hepatic failure×2, TIPS×2, LT×6 |
| Without ductopenia | 1/96 | 1.04% | TIPS×1 |

PBC, primary biliary cholangitis

**Supplementary Table 3**. **Liver-related adverse events according to histologic stages in hospitalized patients with PBC at follow-up**

| **Histological stage** | **Number/Total number of events** | **Prevalence** | **Composition of Liver-Related Adverse Events** |
| --- | --- | --- | --- |
| I | 0/40 | 0% | None |
| II | 3/93 | 3.23% | Hepatic failure×1, TIPS×2 |
| III | 2/48 | 4.17% | LT×2 |
| IV | 6/20 | 30.00% | Hepatic failure×1,TIPS×1, LT×4 |

PBC, primary biliary cholangitis

| **Supplementary Table 4.** Baseline characteristics of patients with early-stage PBC, stratified by ductopenia status | | | | | |
| --- | --- | --- | --- | --- | --- |
| **Characteristics** | **Overall PBC cases**  **(n=298)** | **PBC with ductopenia**  **(n = 74)** | **PBC without ductopenia (n =224)** | **T/**  **χ^2^/Z -value** | ***P* -value** |
| Female | 264  (88.59%) | 64  (86.49%) | 200  (89.28%) | 0.431 | 0.511 |
| Age (years, ‾X±s) | 49.99±10.07 | 49.39±9.82 | 50.18±10.16 | 0.586 | 0.559 |
| IgA (g/L) | 2.86(2.20,3.52) | 2.93(2.03,3.56) | 2.84(2.21,3.52) | -0.561 | 0.575 |
| IgG (g/L) | 16.13±5.58 | 16.28±8.66 | 16.08±4.11 | -0.277 | 0.782 |
| IgM (g/L) | 2.79(1.65,3.82) | 3.22(1.83,3.89) | 2.65(1.53,3.70) | -2.055 | 0.040 |
| PT (s) | 16.03±2.29 | 16.05±2.30 | 16.02±2.28 | -0.132 | 0.895 |
| PTA (%) | 103.50±15.10 | 103.60±17.10 | 103.40±14.50 | -0.102 | 0.919 |
| TBIL  (μmol/L) | 15.60 (11.55,22.10) | 19.30 (13.20,36.30) | 14.60 (11.10,10.40) | -3.861 | <0.001 |
| DBIL  (μmol/L) | 5.70(3.60,12.50) | 6.85(3.80,19.10) | 5.30(3.50,10.50) | -2.301 | 0.021 |
| CHE (U/L) | 6679.00±2023.00 | 6207.00±1722.00 | 6835.00±2093.00 | 2.332 | 0.020 |
| ALT (U/L) | 43.00 (22.00,67.00) | 48.00(23.00,78.00) | 43.00(22.00,63.00) | -0.901 | 0.368 |
| AST (U/L) | 41.00(27.00,62.00) | 47.50(28.00,78.00) | 39.00(27.00,58.00) | -1.310 | 0.190 |
| TBA (U/L) | 10.80(4.70,26.00) | 19.00(7.60,57.90) | 9.70(4.40,22.20) | -3.248 | 0.001 |
| ALB (g/L) | 42.24±6.05 | 40.25±5.34 | 42.90±6.14 | 3.316 | 0.001 |
| GGT (U/L) | 123.50 (54.00,263.50) | 226.00 (74.50,384.50) | 107.50 (51.00,207.50) | -3.439 | <0.001 |
| ALP (U/L) | 139.50 (93.00,218.00) | 195.00 (101.50,283.50) | 132.50 (91.00,189.50) | -2.799 | 0.005 |
| NEUT (×10^9^/L) | 2.99(2.40,3.80) | 2.90(2.26,3.79) | 3.09(2.42,33.81) | -0.850 | 0.396 |
| WBC (×10^9^/L) | 5.83±1.94 | 5.36±1.69 | 5.99±1.99 | 2.444 | 0.015 |
| RBC (×10^12^/L) | 4.24±0.57 | 4.06±0.59 | 4.31±0.55 | 3.343 | <0.001 |
| PLT (×10^9^/L) | 223.00±78.00 | 209.00±81.00 | 227.00±76.00 | 1.732 | 0.084 |
| HGB (g/L) | 126.68±18.26 | 122.10±18.60 | `128.20±17.95 | 2.522 | 0.012 |
| ANA antibody |  |  |  | 8.700 | 0.003 |
| Non-Strong Positive (<1:1000) | 184 (61.74%) | 35 (47.30%) | 149 (66.51%) | 19.725 | <0.001 |
| Strong Positive (≥1:1000) | 114 (38.26%) | 39 (52.70%) | 75 (33.48%) |  |  |
| AMA antibody |  |  |  | 2.199 | 0.138 |
| Non-Strong Positive (<1:1000) | 139 (46.64%) | 29 (39.19%) | 110 (49.11%) |  |  |
| Strong Positive (≥1:1000) | 159 (53.36%) | 45 (60.81%) | 114 (50.89%) |  |  |
| Anti-gp210 antibody |  |  |  | 2.369 | 0.124 |
| Non-Strong Positive (-/+/++/+++) | 271 (90.93%) | 64 (86.49%) | 207 (92.41%) |  |  |
| Strong Positive (++++) | 27 (9.06%) | 10 (13.51%) | 17 (7.59%) |  |  |
| AMA-M2 antibody |  |  |  | 0.685 | 0.408 |
| Non-Strong Positive (＜800 RU/mL) | 228  (76.51%) | 54  (72.97%) | 174  (77.67%) |  |  |
| Strong Positive (≥800 RU/mL) | 70  (34.49%) | 20  (27.03%) | 50  (22.32%) |  |  |

PBC, primary biliary cholangitis; IgA, immunoglobulin A; IgG, immunoglobulin G; IgM, immunoglobulin M; PT, prothrombin time; PTA%, prothrombin time activity; TBIL, total bilirubin; DBIL, direct bilirubin; CHE, cholinesterase; GGT, gamma-glutamyl transferase; ALT, alanine aminotransferase; AST, aspartate aminotransferase; TBA, total bile acid; ALB, albumin; ALP, alkaline phosphatase; WBC, white blood cell; RBC, red blood cell, NEUT, neutrophil count; PLT, platelet count; HGB, hemoglobin; ANA, antinuclear antibody; AMA, antimitochondrial antibody.

**Supplementary Table 5.** Univariate and multivariate logistic regression analysis to identify baseline factors associated with ductopenia in patients with early-stage PBC

|  | **Univariate analysis** | | **Multivariate analysis** | |
| --- | --- | --- | --- | --- |
| **Biological markers** | **Odds ratio (95% CI)** | ***P*-value** | **Odds ratio (95% CI)** | ***P*-value** |
| IgM(g/L) | 1.128 (1.002–1.269) | 0.046 | - | - |
| ALP (U/L) | 1.002 (1.000–1.003) | 0.011 | - | - |
| CHE (U/L) | 1.000 (1.000–1.000) | 0.022 | - | - |
| TBA (U/L) | 1.009 (1.003–1.015) | 0.003 | - | - |
| ALB(g/L) | 0.921 (0.876–0.969) | 0.001 | 0.943 (0.896–0.993) | 0.025 |
| TBIL(μmol/L) | 1.028 (1.014-1.044) | ＜0.001 | 1.020 (1.005–1.035) | 0.007 |
| ANA antibody (≥1:1000) | 2.214 (1.298, -3.776) | 0.008 | 2.168 (1.224–3.837) | 0.008 |
| GGT (U/L) | 1.002 (1.001–1.003) | 0.005 | 1.002 (1.001–1.003) | 0.005 |
|  | | | | |

**B**


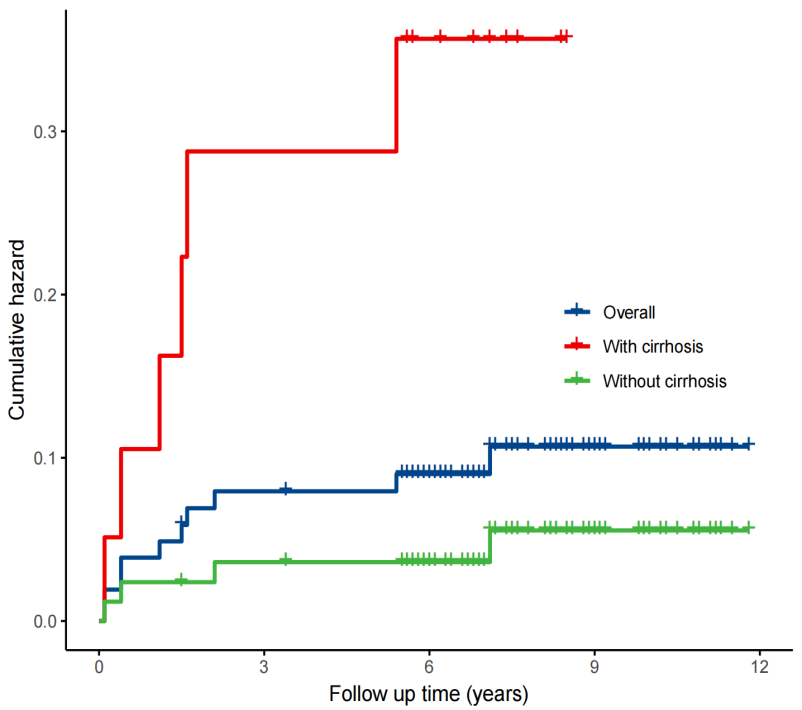

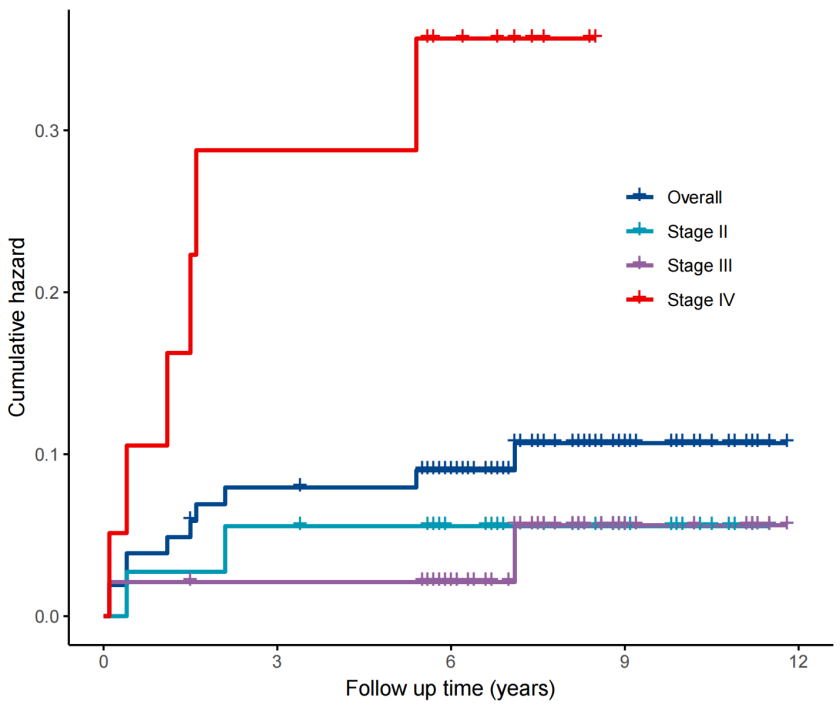


**A**

**Supplementary Figure4.**Cumulative Hazard of Liver-Related Adverse Events by in Primary Biliary Cholangitis with Ductopenia. Stratification by (A) Stages, (B) with cirrhosis.


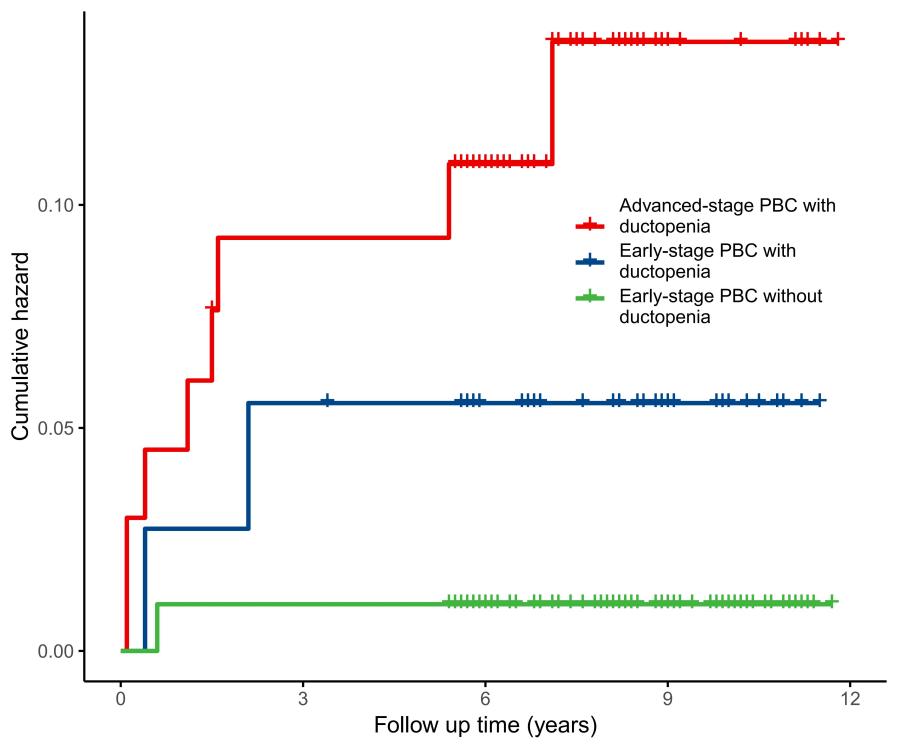


**Supplementary Figure5.**Cumulative Hazard of Liver-Related Adverse Events by in Primary Biliary Cholangitis. Stratification by early-stage PBC without ductopenia, early-stage PBC with ductopenia, advanced -stage PBC with ductopenia.
